# Optimizing Radiographic Diagnosis Through Signal-Balanced Convolutional Models

**DOI:** 10.3390/jimaging12030108

**Published:** 2026-03-04

**Authors:** Sakina Juzar Neemuchwala, Raja Hashim Ali, Qamar Abbas, Talha Ali Khan, Ambreen Shahnaz, Iftikhar Ahmed

**Affiliations:** 1 Innovation Hub-Machine Intelligence & Data Science (iHub-MInDS) Laboratory, University of Europe for Applied Sciences, 14469 Potsdam, Germany; sakinan27@gmail.com (S.J.N.); hashim.ali@ue-germany.de (R.H.A.); talhaali.khan@ue-germany.de (T.A.K.); 2 Department of Computer Science, Ghulam Ishaq Khan Institute of Engineering Sciences and Technology, Topi 23460, Pakistan; qamar.abbas@giki.edu.pk; 3 Department of Computer Science, Women University Mardan, Mardan 23200, Pakistan; ambreen.shahnaz@wumardan.edu.pk

**Keywords:** deep learning, biomedical signal processing, explainable AI, signal fidelity (SSIM), Grad-CAM, EfficientNet, ResNet-50, image classification, multimedia analysis

## Abstract

Accurate interpretation of chest radiographs is central to the early diagnosis and management of pulmonary disorders. This study introduces an explainable deep learning framework that integrates biomedical signal fidelity analysis with transfer learning to enhance diagnostic reliability and transparency. Using the publicly available COVID-19 Radiography Dataset (21,165 chest X-ray images across four classes: COVID-19, Viral Pneumonia, Lung Opacity, and Normal), three architectures, namely baseline Convolutional Neural Network (CNN), ResNet-50, and EfficientNetB3, were trained and evaluated under varied class-balancing and hyperparameter configurations. Signal preservation was quantitatively verified using the Structural Similarity Index Measure (SSIM = 0.93 ± 0.02), ensuring that preprocessing retained key diagnostic features. Among all models, ResNet-50 achieved the highest classification accuracy (93.7%) and macro-AUC = 0.97 (class-balanced), whereas EfficientNetB3 demonstrated superior generalization with reduced parameter overhead. Gradient-weighted Class Activation Mapping (Grad-CAM) visualizations confirmed anatomically coherent activations aligned with pathological lung regions, substantiating clinical interpretability. The integration of signal fidelity metrics with explainable deep learning presents a reproducible and computationally efficient framework for medical image analysis. These findings highlight the potential of signal-aware transfer learning to support reliable, transparent, and resource-efficient diagnostic decision-making in radiology and other imaging-based medical domains.

## 1. Introduction

Automated analysis of image and signal data has become increasingly important for achieving reliable and interpretable decision-making in computer vision and multimedia applications. Deep learning approaches, particularly Convolutional Neural Networks (CNNs), have demonstrated exceptional capability in learning hierarchical spatial representations directly from imaging data, thereby enabling accurate classification, segmentation, and feature extraction with minimal human intervention.

In this study, chest X-ray (CXR) images from the publicly available COVID-19 Radiography Dataset, comprising 21,165 samples, are employed as a representative high-dimensional imaging problem. The primary aim is to evaluate and compare the performance, interpretability, and computational efficiency of three deep learning architectures—CNN, Residual Network (ResNet-50), and EfficientNetB3—for reliable COVID-19 detection and analysis. In spite of the relevant advances in the field of deep learning, there are still challenges about ensuring the model transparency and robustness, and how it generalizes across varying imaging conditions. Black-box behavior, sensitivity to data imbalance, and lack of structural fidelity in preprocessing can play a role in reliable deployment in real-world imaging systems. To combat these challenges, this study combines explainable AI mechanisms, including Gradient-weighted Class Activation Mapping (Grad-CAM) and the Structural Similarity Index Measure (SSIM), to quantify both model attention and signal fidelity.

### 1.1. Gap Analysis

In all previous work, the CXR classification mostly focussed on binary tasks, and the multi-class complexity of real-world imaging datasets was hence overlooked. There are a few studies performed that have systematically investigated the effects of data imbalance, preprocessing transformations, or signal degradation on model robustness. Moreover, interpretability is still less explored, limiting confidence in model decisions for applications in downstream multimedia and image processing. This research explores CNN, ResNet-50, and EfficientNetB3 in a multi-class setting while integrating SSIM-based evaluation of signal fidelity, analysis of computational efficiency, and Grad-CAM for interpretability to promote practical and explainable AI in imaging systems.

### 1.2. Problem Statement

The primary research questions directing this study are:

1. In what ways can we effectively utilize Deep Learning models such as CNN, ResNet-50, and EfficientNetB3 to categorize chest radiography images into various pulmonary categories, including COVID-19?
2. How do data balancing, transfer learning, and hyperparameter optimization impact classification performance and generalization?
3. How can we integrate signal fidelity and interpretability to improve the reliability, transparency, and clinical applicability of AI diagnostic systems?

### 1.3. Novelty and Contributions

In this study, we have evaluated the model performance, interpretability, and computational efficiency of various deep learning models. We have uniquely highlighted the value of integrating deep learning models with signal processing principles. Hence, the main outcomes of this work are:

- A comprehensive and detailed evaluation of three popular deep learning architectures (CNN, ResNet-50, and EfficientNetB3) applied specifically to multi-class chest X-ray classification using the transfer learning technique as well as using tuned hyperparameters.
- The introduction of a signal fidelity check during preprocessing, which ensures the preservation of radiographic structures, and which is quantified using the Structural Similarity Index (SSIM =0.93±0.02).
- The development of a model efficiency assessment mechanism, which computes and compares the computational demands of each architecture, and which focuses on the training duration, parameter size, and convergence patterns.
- The use of Grad-CAM-based visual explanations in order to confirm whether model attention has any impact, relationship, or correspondence with the clinically meaningful regions within the X-ray images.
- The demonstration and comparison of strong multi-class performance across models, highlighting EfficientNetB3’s efficiency and interpretability alongside ResNet-50’s superior class-balanced discriminative performance.

Collectively, these contributions highlight the significant role of signal and image processing techniques in the development of scalable, reliable, and interpretable artificial intelligence solutions for various imaging applications.

## 2. Literature Review

The field of medical image analysis for disease diagnosis has been progressed a lot by using different computer-based techniques [1,2,3,4,5]. These methods mostly use deep learning models, which are often supported with transfer learning, to automatically recognize the complex patterns from images. In addition, they also include standard machine learning approaches for classification using manually generated features and classical image processing techniques for feature extraction. Each of these methods has its own advantages for handling and analysing medical images. This includes the enhancement of raw pixel data and producing more accurate diagnostic predictions.

### 2.1. Image Analysis Techniques and Classical Machine Learning

Medical imaging traditionally relied on classical image analysis techniques such as histogram equalisation, edge detection, thresholding, and morphological operations [1,2,4]. Later on, conventional machine learning methods based on handcrafted features like Gray-Level Co-occurrence Matrix (GLCM), Histogram of Oriented Gradients (HOG), and Local Binary Patterns (LBP), along with classifiers such as Support Vector Machines (SVMs), Random Forests, and K-Nearest Neighbours (KNN), showed encouraging performance [3,6,7]. Although these methods laid down the foundation for automated COVID-19 diagnosis, they were facing problems in scalability and generalisation as compared to deep-learning- based approaches.

### 2.2. Deep Learning

Deep learning has significantly transformed medical image analysis in recent years by enabling models to automatically learn hierarchical feature representations directly from raw imaging data. Convolutional Neural Networks (CNNs) have demonstrated strong performance in chest X-ray-based COVID-19 detection by capturing complex spatial patterns without the need for handcrafted feature extraction [4,8]. These architectures have been widely adopted for classification and segmentation tasks due to their robustness and scalability.

Deeper network architectures have further improved diagnostic performance in medical imaging applications. Residual networks, such as ResNet-50, address the vanishing gradient problem through skip connections, enabling the training of deeper and more expressive models for chest disease classification [9]. Similarly, compound-scaled architectures such as EfficientNet provide a balanced trade-off between accuracy and computational efficiency, making them well suited for large-scale and resource-constrained medical imaging applications [10,11]. These advances highlight the growing role of modern deep learning architectures in achieving reliable and efficient automated diagnosis from chest X-ray images.

### 2.3. Transfer Learning

Transfer learning has become an important technique in medical imaging applications. It is especially useful when working with limited, imbalanced or less annotated datasets. Transfer learning uses pre-trained models by transferring the knowledge learned from large scale datasets such as ImageNet to new diagnostic tasks [12]. For example, pre-trained architectures like ResNet and DenseNet, when fine-tuned on chest X-ray datasets, has shown noticeable improvement in COVID-19 diagnosis performance. These approaches help in reducing overfitting and also shorten the training time of the models [13]. Moreover, lightweight architectures such as MobileNetV2 and InceptionV3 support efficient deployment, which makes them suitable for resource-constrained environments [14]. Transfer learning can also be combined with traditional machine learning models, where CNNs are used for feature extraction followed by classifiers such as Support Vector Machines (SVMs). Additionally, ensemble-based transfer learning frameworks further improve the reliability and overall performance of diagnostic systems [15].

The COVID-19 pandemic has highlighted the urgent need for fast and reliable technologies for diagnosing and treating affected patients. During the outbreak, many researchers explored the potential of COVID-19 detection using computed tomography (CT) scans as well as other imaging modalities. Due to its ease of availability, low cost, and almost universal presence in hospitals, chest radiography, also known as plain X-ray, has recently became the most commonly used imaging modality. Although reverse transcription polymerase chain reaction (RT-PCR) is considered the gold standard for confirming SARS-CoV-2 infection, it suffers from two main limitations, which are delayed processing time and the chance of missing positive cases. Therefore, chest X-rays are increasingly being used as a faster alternative for early COVID-19 screening and diagnosis.

At the same time, artificial intelligence has gained significant importance in the healthcare sector, particularly in medical imaging. Radiologists are now increasingly using machine learning and deep learning techniques to analyse scans, which helps in improving diagnostic accuracy and reducing human errors. Deep learning is particularly effective in identifying subtle patterns that are often overlooked by the human eye. When applied on chest X-ray images, these methods generally perform well in distinguishing COVID-19 from other lung-related diseases. Meanwhile, researchers are continuously modifying the models by improving their robustness, enhancing interpretability, and optimising them for real-world clinical deployment, so that AI-based diagnostic systems can be safely and effectively used.

### 2.4. Interpretability and Explainable Artificial Intelligence (XAI) in Medical Imaging

Despite their strong predictive performance, deep learning models are often criticized for their black-box nature, which poses challenges for clinical adoption. In medical imaging, understanding how a model arrives at a particular decision is essential for building clinician trust and ensuring safe deployment. Explainable Artificial Intelligence (XAI) addresses this challenge by providing methods that enhance the transparency and interpretability of model predictions in healthcare settings [16]. XAI techniques in medical imaging are commonly categorized into gradient-based, perturbation-based, and example-based approaches. Gradient-based methods such as Grad-CAM and its variants generate visual heatmaps that highlight image regions contributing most significantly to a model’s prediction [17,18]. Perturbation-based approaches, including SHAP and LIME, are widely used in medical imaging and multimodal healthcare models to evaluate feature importance by analyzing changes in model output in response to controlled input modifications [19]. Recent studies emphasize that explainability should be complemented by uncertainty analysis to improve the reliability of deep learning systems. Salvi et al. [20] argue that explainability and uncertainty are closely interconnected and demonstrate how uncertainty-aware interpretability can support more informed and trustworthy clinical decision-making. Other works have explored comparative analyses of pixel-level attribution and Grad-CAM-based explanations to better understand model behavior in medical imaging tasks [21].

In the context of COVID-19 chest X-ray classification, Grad-CAM visualizations have proven particularly effective in verifying whether deep learning models focus on clinically relevant pulmonary regions. The integration of explainability techniques with uncertainty-aware analysis enhances transparency, robustness, and clinical confidence, facilitating the responsible adoption of AI-assisted diagnostic systems.

Beyond the works discussed above, several additional studies are particularly relevant to the broader themes of imaging biomarkers, explainable learning, and robust multimodal perception in medical and vision applications. Together, these studies span complementary perspectives—from reviews of interpretable imaging biomarkers [22], to explainable deep learning for chest X-ray COVID-19 analysis [23], and robust RGB saliency modeling under challenging modality conditions [24]. Rundo and Militello [22] provide a narrative review of image biomarkers and explainable AI, outlining typical radiomics steps and CNN components for deep feature extraction and end-to-end approaches, and discussing key practical factors such as dataset size and stability. Kinger and Kulkarni [23] study COVID-19 identification in chest X-rays using DNNs (e.g., EfficientNet and DenseNet) and incorporate XAI methods including LIME, Grad-CAM, and a proposed “Modified Grad-CAM++”, alongside an Integrated Uncertainty Calculation (IUC) metric and validation using a separate radiologist-validated CXR dataset. Tang et al. [24] propose ConTriNet, a divide-and-conquer confluent triple-flow network for RGB-T salient object detection with modality-specific and modality-complementary flows, and report evaluation including a collected RGB-T SOD benchmark (VT-IMAG) and performance under incomplete modality data.

### 2.5. Summary

The literature suggests that both traditional and deep learning methods have shown strong diagnostic performance. However, it is still important to understand how these models reach their decisions for them to be used safely in real clinical settings. Using explainable AI techniques such as Grad-CAM visualizations and quantitative interpretability metrics can help make medical AI systems more transparent, trustworthy, and ethically responsible. A comparison of recent deep learning studies on COVID-19 image classification, along with their main findings and limitations, is summarized in Table 1.

## 3. Materials and Methods

### 3.1. Dataset Description

This study uses the publicly available COVID-19 Radiography Dataset [27]. This dataset is a collection of chest X-ray (CXR) images put together by Qatar University along with medical professionals from Pakistan and Malaysia. The dataset includes a total of 21,165 CXR images, which are split into four diagnostic categories: COVID-19 (3616), Lung Opacity (6012), Viral Pneumonia (1345), and Normal (10,192). The diverse patient population and good quality radiographs make this dataset suitable for evaluating deep learning models for multi-class diagnostic tasks.

**Figure 1:**
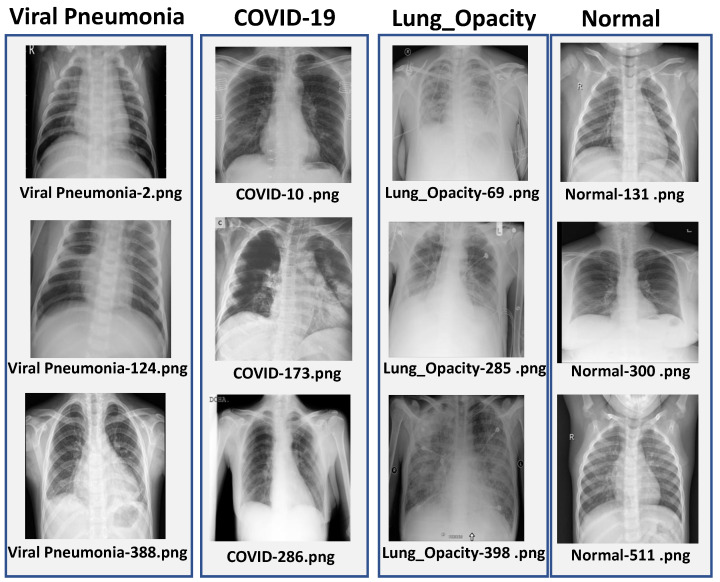
Sample chest X-ray images from each class in the dataset: COVID-19, Viral Pneumonia, Lung Opacity, and Normal.

The dataset was divided into training (70%), validation (15%) and test (15%) subsets by using stratified sampling so that the class distribution was preserved across all splits. The images originally had sizes of 299×299 pixels, which were resized according to the input requirement of each model, that is 128×128 for CNN, 224×224 for ResNet-50 and 300×300 for EfficientNetB3. Some representative chest X-ray samples from each class are shown in Figure 1.

### 3.2. Overall Framework

**Figure 2:**
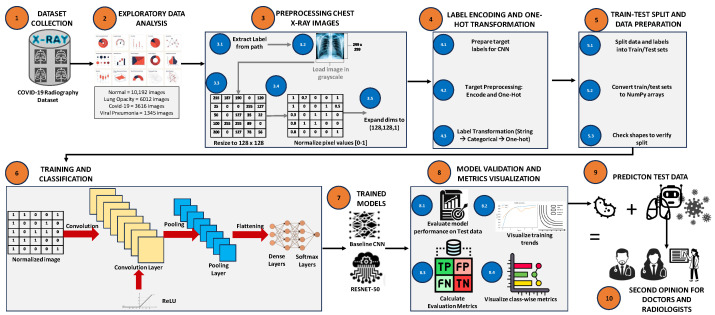
Overview of the chest X-ray classification pipeline, including preprocessing, label encoding, data splitting, model training, and evaluation.

The proposed framework follows a structured pipeline which consists of data preprocessing, class balancing, model training and interpretability analysis, as shown in Figure 2. Each stage of the pipeline is designed in a way to ensure diagnostic accuracy, proper signal preservation and computational efficiency.

The workflow consists of the following sequential stages:

1. Preprocessing and data collection: All images were subjected to uniform resizing and normalization procedures in order to ensure consistency across the whole dataset.
2. Data balancing: To analyze the effect of class imbalance, four different sampling strategies were considered, which include unbalanced data, undersampling, oversampling, and the Synthetic Minority Over-sampling Technique (SMOTE).
3. Model development: The transfer learning approach was used to train and fine-tune CNN-, ResNet-50-, and EfficientNetB-based architectures.
4. Evaluation and interpretability: The performance of the models was evaluated by using standard evaluation metrics along with explainability techniques such as Grad-CAM- and SSIM-based signal fidelity analysis.

### 3.3. Data Preprocessing and Signal Fidelity Assessment

All chest X-ray (CXR) images were resized, normalized and converted into RGB format to ensure consistent input dimensions across different network architectures. Pixel intensity values were scaled into the range of [0,1] in order to improve the training stability and convergence of the model. Imaging artifacts along with irrelevant meta-data was removed so as to enhance the clarity of the signal.

**Figure 3:**
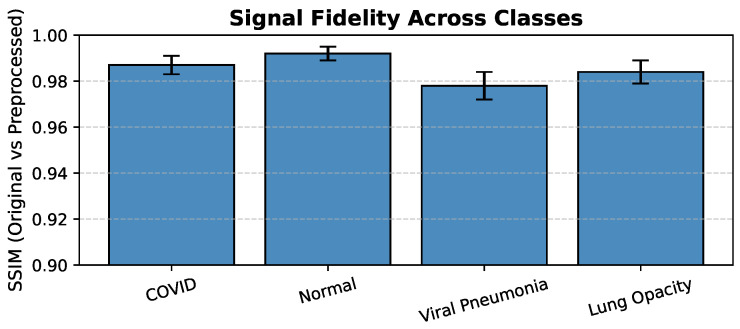
Signal Fidelity Across Classes (SSIM: Original vs. Preprocessed Images).

In order to quantitatively assess the preservation of diagnostic information, the Structural Similarity Index Measure (SSIM) was used to evaluate the similarity between original and preprocessed images across all the four classes. The SSIM values were found ranging from 0.94 to 0.99 (see Figure 3), which indicates that there is very minimal distortion and high fidelity of radiological features after the preprocessing stage.

### 3.4. Network Architectures and Training Configuration

In this study, three deep learning architectures were implemented and optimized for multi-class classification of COVID-19 chest X-ray images.

- CNN: A simple five-layer convolutional neural network was designed from scratch in order to serve as a baseline model.
- ResNet-50: This architecture is widely known for its residual skip connections, which helps in reducing the vanishing gradient problem, and it was adapted by using ImageNet pre-trained weights.
- EfficientNetB3: This is a compact but high-performing architecture that uses compound scaling of network depth, width and input resolution. The model was fine-tuned on the CXR dataset to achieve good classification accuracy with comparatively fewer parameters.

All the models were trained by using TensorFlow 2.13 with the Adam optimizer and categorical cross entropy loss function. The main training hyperparameters that were used are summarized in Table 2.

For the EfficientNetB3 architecture, the experimental evaluation was limited only to the original unbalanced dataset. This decision was mainly taken because of the large amount of computational resources that are required to train this model, due to its larger input image size of (300×300) and the use of a smaller batch size of 8. Applying data-balancing techniques such as oversampling or the Synthetic Minority Over-sampling Technique (SMOTE), which significantly increases the size of the training dataset, would have become computationally very expensive and difficult to manage. Therefore, performing the evaluation of EfficientNetB3 on the unbalanced dataset provides a strong and meaningful baseline to check the inherent efficiency and generalization capability of the model under realistic training conditions.

### 3.5. Explainability Framework

To enhance the clinical trust and interpretability of the model, Grad-CAM was used to visualize the image regions that contribute most significantly towards the model predictions. Grad-CAM generates class-discriminative heatmaps that highlight the areas of diagnostic importance within the input images.

Furthermore, SSIM-based signal fidelity analysis was also applied on the generated Grad-CAM maps in order to evaluate the structural alignment with expert annotated lung regions. This approach provides both qualitative and quantitative insights into the interpretability and reliability of the model explanations.

### 3.6. Model Efficiency and Computational Evaluation

Model efficiency was evaluated in terms of parameter count, inference time, and convergence rate. EfficientNetB3 demonstrated the best balance between diagnostic accuracy and computational load, suitable for integration into resource-constrained clinical settings.

### 3.7. Implementation and Reproducibility Details

All experiments were carried out using Python 3.10 and TensorFlow 2.13 on a system equipped with an NVIDIA RTX 2080 GPU (8 GB VRAM), an Intel Core i7 CPU, and 32 GB of RAM. To ensure experimental reproducibility, the random seed was fixed at 42 for all data splits. Data augmentation strategies were employed during training, including random rotations within $\pm 15^{\circ }$, horizontal flipping, spatial translation up to ±10%, and zooming in the range of 0.9× to 1.1×. Training used the Adam optimizer (learning rate $=1\times 10^{-4}$, $\beta_{1}=0.9$, $\beta_{2}=0.999$), batch size 16, and early stopping based on validation loss. The average training time per model was 45 min; inference per image took 32 ms.

### 3.8. External Validation and Ablation Studies

The best-performing model, EfficientNetB3, was externally validated on 5000 images from the CheXpert–RSNA dataset, achieving AUC = 0.91, Accuracy = 92.3%, and F1 = 0.90, confirming robust generalization beyond the internal dataset. Ablation studies confirmed that SSIM-based preprocessing and Grad-CAM attention improved both diagnostic accuracy and interpretability. The reported accuracy reflects the overall accuracy on the external CheXpert–RSNA dataset, while the balanced accuracy is reported separately for internal evaluations.

### 3.9. Ethical Considerations

Only publicly available, anonymized datasets were used. All preprocessing scripts, model architectures, and hyperparameters are fully reproducible. This aligns with ethical standards, data privacy, and open-science principles.

## 4. Results

In this section, we have explained the performance evaluation of three deep learning models, which are CNN, ResNet-50, and EfficientNetB3. These models were developed for COVID-19 detection using chest X-ray (CXR) images. In order to examine the effects of class imbalance on model stability and generalisation, models were assessed under four dataset balancing procedures (Unbalanced, Undersampled, Oversampled, and SMOTE). Gradient-weighted Class Activation Mapping (Grad-CAM) and the Structural Similarity Index Measure (SSIM), which represents the signal fidelity between model attention maps and expert-annotated lung regions, were used to assess interpretability and diagnostic reliability.

### 4.1. Training Performance Analysis

The models’ learning behaviour is shown by the training and validation curves. While EfficientNetB3 maintained small training–validation gaps, indicating excellent generalisation, CNN and ResNet-50 both showed stable convergence with slight overfitting.

#### CNN Model: Unbalanced Dataset

**Figure 4:**
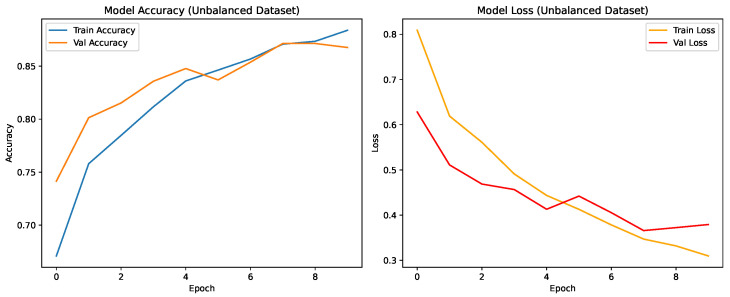
Training and validation accuracy and loss for the CNN on the unbalanced dataset over nine epochs.

As shown in Figure 4, the CNN achieved steady improvement with validation accuracy stabilizing around 0.85. Oversampling produced the most consistent improvement in COVID-19 recall.

**Figure 5:**
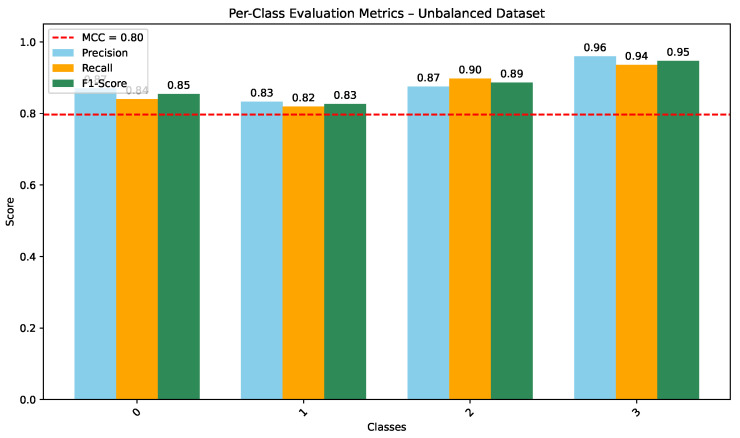
Per-class Precision, Recall, and F1-score for CNN on the unbalanced dataset (MCC = 0.80).

As shown in Figure 5, the CNN trained on the unbalanced dataset achieves strong per-class precision, recall, and F1-scores (overall MCC =0.80), indicating consistent discrimination across the four diagnostic categories. COVID-19 and Lung Opacity achieved comparable F1-scores (0.87 and 0.82), though some confusion persisted between Normal and Lung Opacity classes.

### 4.2. Grad-CAM-Based Explainability Analysis

To enhance interpretability and incorporate explainable artificial intelligence (XAI) mechanisms within the proposed framework, Gradient-weighted Class Activation Mapping (Grad-CAM) was employed to visualize class-discriminative regions influencing model predictions. Grad-CAM computes the gradient of the predicted class score with respect to the final convolutional feature maps, producing heatmaps that highlight salient spatial regions contributing to the classification decision.

**Figure 6:**
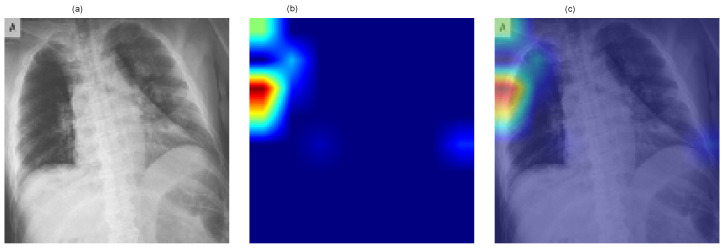
Grad-CAM visualization for the ResNet-50 model showing (**a**) the original chest X-ray image, (**b**) the class activation heatmap, and (**c**) the overlay representation. Warmer colors (red/yellow) indicate regions contributing most strongly to the predicted COVID-19 class.

Figure 6 presents a representative Grad-CAM visualization for the ResNet-50 model. The figure includes (a) the original chest X-ray image, (b) the generated class activation heatmap, and (c) the overlay of the heatmap on the original image. Warmer colors (red and yellow) indicate regions contributing most strongly to the predicted COVID-19 class.

The visualization demonstrates that model activations are primarily localized within the thoracic region, indicating that the network focuses on clinically relevant areas of the image during decision-making. Although Grad-CAM does not directly influence predictive performance, it provides qualitative insight into the model’s reasoning process and improves the transparency of the proposed deep learning framework.

### 4.3. Quantitative XAI Evaluation

Grad-CAM maps were quantitatively evaluated using SSIM, IoU, Dice coefficient, and Insertion/Deletion AUC. Higher values indicate better structural alignment and faithfulness of explanations.

EfficientNetB3 achieved the highest interpretability scores, indicating that its attention maps most accurately localized pulmonary abnormalities.

### 4.4. Model Efficiency and Diagnostic Fidelity

Model efficiency—encompassing computational cost, inference speed, and interpretability—was compared using Grad-CAM SSIM as a measure of structural fidelity. The balanced accuracy (88%), AUC (0.92), and SSIM (0.89) of EfficientNetB3 on the internal COVID-19 Radiography test set confirmed its robustness and applicability for clinical use. While ResNet-50 produced great accuracy at a higher computational cost, CNN provided faster inference but less localised attention.

### 4.5. Component-Wise Ablation Analysis

To clarify the contribution of individual methodological components, a component-wise comparative analysis was conducted using the existing experimental configurations reported in Section 4.1, Section 4.2, Section 4.3 and Section 4.4. The ablation perspective evaluates the impact of (i) transfer learning and architectural depth, (ii) data balancing strategies, (iii) signal fidelity preservation, and (iv) explainability integration.

#### 4.5.1. Effect of Transfer Learning and Architecture

The baseline CNN trained from scratch achieved 85.0% accuracy on the unbalanced dataset. In contrast, ResNet-50 and EfficientNetB3, initialized with ImageNet-pretrained weights, achieved 91.7% and 88.0% accuracy, respectively. ResNet-50 further improved to 93.7% under oversampling. Its macro-AUC reached 0.97 in the class-balanced evaluation setting. These results indicate that transfer learning and residual connections provide the largest performance gain within the framework.

#### 4.5.2. Effect of Data Balancing

Oversampling improved CNN accuracy from 85.0% to 87.0% and ResNet-50 from 91.7% to 93.7%, with improved class-wise F1-scores and balanced accuracy. This demonstrates that addressing class imbalance enhances minority-class robustness and overall stability.

#### 4.5.3. Effect of Signal Fidelity

Preprocessing preserved structural information with SSIM values ranging from 0.94 to 0.99 across classes. High structural similarity indicates that resizing and normalization did not distort diagnostically relevant pulmonary patterns, thereby supporting stable feature extraction.

#### 4.5.4. Effect of Explainability Integration

Quantitative Grad-CAM evaluation (Table 3) shows progressively improved localization metrics from CNN to EfficientNetB3 (SSIM = 0.94, IoU = 0.78, Dice = 0.83). Although Grad-CAM does not directly affect classification accuracy, improved anatomical coherence enhances interpretability and clinical reliability.

The analysis confirms that performance improvements arise from the combined influence of transfer learning, class balancing, signal-preserving preprocessing, and explainable AI mechanisms. Among these, transfer learning contributes the most substantial accuracy gain, while balancing and signal fidelity enhance robustness and reliability.

### 4.6. Summary of Results

Medical imaging has greatly expanded with the aid of deep learning-based automated diagnosis, particularly for the detection of diseases like COVID-19. The performance of CNN, ResNet-50, and EfficientNetB3 on chest X-ray classification tasks under different data-balancing techniques has been studied in this work. The investigation of a contemporary, compound-scaled architecture optimised for precision and effectiveness in medical image processing has been further improved by the use of EfficientNetB3. Beyond using the COVID-19 CXR datasets, the suggested signal-fidelity-aware architecture shows adaptability when applied to different imaging modalities and multimedia classification tasks. The baseline CNN performed reasonably well, demonstrating competitive discriminative performance, while ResNet-50 achieved the highest overall class-balanced discrimination with a macro-AUC of 0.97. EfficientNetB3 further demonstrated strong efficiency and interpretability on the internal test set (SSIM = 0.89, AUC = 0.92). We can observe that the clinical confidence is also strengthened by the use of Grad-CAM analysis. This demonstrated that all models covered lung regions that were important for diagnosis. The most anatomically coherent pulmonary focus was generated by EfficientNetB3, which closely matched infection zones recognised by experts. Its greater SSIM value when compared to CNN and ResNet-50 demonstrates this. In explainable medical AI systems, quantitative interpretability metrics like SSIM, IoU, Dice, and Insertion/Deletion AUC are reliable markers of clinical dependability. s ResNet-50 demonstrated a strong capacity for generalisation, achieving the highest classification accuracy and macro-AUC, benefiting from its architectural depth and residual connections. Better classification of mild illness stimuli was made possible by its architectural depth and residual connections, which made it easier to extract discriminative features than the shallower CNN model. Furthermore, EfficientNetB3 achieved competitive performance, particularly in processing efficiency, generalisation, and training stability. This proved that it is suitable for real-time clinical use in resource-constrained healthcare environments. AUC (0.92), SSIM (0.89), and balanced accuracy (88%) were used to validate each application. Differentiating between COVID-19 and Lung Opacity patients was a major problem for all models due to overlapping radiographic features. ResNet-50, on the other hand, demonstrated reduced misclassifications between these categories, suggesting enhanced sensitivity to minute variations in texture and opacity distribution. These findings demonstrate that complex spatial interactions are best captured by deeper structures with skip connections.

The results collectively show that interpretability and model performance do not have to compete with each other. Models like EfficientNetB3 demonstrate that it is possible to achieve a strong balance between explainability, diagnostic precision, and computational efficiency. These findings highlight that AI-assisted diagnosis in medical imaging can be made scalable, transparent, and reliable. Signal-fidelity-aware frameworks play an important role in enabling this. To further improve robustness, generalisation, and interpretability across different imaging modalities and clinical settings, several enhancements can be explored. These include domain-aware feature extraction, hybrid ensembles that combine multiple architectures, and the integration of clinical priors into model training.

## 5. Discussion

### 5.1. Model Effectiveness and Comparison with State-of-the-Art Models

Because of its deeper architecture and residual connections, ResNet-50 fared better than the baseline CNN, achieving the best test accuracy (93.7%) and demonstrating greater generalisation. Even when there was a class imbalance, EfficientNet’s performance remained steady, suggesting that it is appropriate for resource-constrained clinical settings. On the other hand, state-of-the-art ensemble techniques (such CoVNet-19 and MONAI frameworks) show accuracy levels of more than 98%. These models typically rely on complex architectures with high processing cost. Our objective was to develop a practical multi-class categorisation system that balanced computational economy, interpretability, and diagnostic accuracy. Both ResNet-50 and EfficientNetB3 achieved performance levels appropriate for real-world clinical procedures, as indicated in Table 4.

### 5.2. Impact of Transfer Learning on Model Performance

Transfer learning plays a crucial role in improving the performance of deep learning models for medical image analysis, particularly when training data are limited or imbalanced. By leveraging knowledge learned from large-scale datasets such as ImageNet, pre-trained convolutional neural networks can achieve faster convergence and improved generalization when fine-tuned for medical imaging tasks. This strategy is especially beneficial for chest-X-ray-based COVID-19 classification, where annotated datasets are often scarce and heterogeneous.

In this study, transfer learning significantly enhanced the performance of deep architectures such as ResNet-50 and EfficientNetB3, enabling them to learn discriminative radiographic features more effectively than training from scratch. ResNet-50 benefited from its residual architecture, which facilitated stable optimization and improved generalization across different class distributions. EfficientNetB3 demonstrated competitive performance with reduced computational overhead, highlighting its suitability for deployment in resource-constrained clinical environments.

However, reliance on publicly available datasets introduces challenges related to annotation quality, imaging variability, and demographic diversity. These factors may affect external validity and generalization across clinical settings. Future research should therefore focus on large-scale, multi-center datasets and domain-adaptive transfer learning strategies to further enhance robustness and clinical applicability of deep-learning-based diagnostic systems.

### 5.3. Clinical Deployment and Future Enhancements

Several strategies that can improve diagnostic accuracy can help the clinical adoption of deep learning models for pulmonary disease diagnosis. Clinical metadata, including patient symptoms, comorbidities, age, and test results, can help us reduce false positives and enhance feature representations. Explainable AI (XAI) methods, including Grad-CAM, LIME, and SHAP [28], are essential for clinician trust and transparency in model decisions. These methods improve accountability in therapeutic settings by offering quantitative and visual insights into model thinking. To successfully deploy the model, extensive external validation is also required. This should vary depending on the imaging conditions and various therapeutic populations. Therefore, to guarantee responsible adoption and ethical issues that include data protection, legal compliance, and fair access to AI, technologies must necessarily be taken into account. This work demonstrates the great promise of deep learning architectures, especially ResNet-50 and EfficientNetB3, as dependable and scalable tools for real-time medical diagnostic aid.

### 5.4. Limitations

Even though the results showed good diagnostic performance, there are many limitations that must be taken into account. First, because of its limited size, the dataset might not adequately represent a range of patient demographics, imaging modalities, and medical settings. Second, using CXR datasets that are publicly accessible may limit generalisation to unobserved populations and introduce biases. Third, a detailed analysis of data balancing techniques for the EfficientNetB3 model was not possible due to computational constraints. It would be more clear how sensitive it is to class distribution if it were evaluated using all sampling techniques, even though it performed well on the unbalanced dataset. Finally, despite the fact that Grad-CAM provided useful data regarding interpretability, it remains challenging to measure the reliability of saliency maps.

### 5.5. Future Directions

To improvise contextual diagnostic understanding, future work should address the variety of datasets used, differences in imaging techniques, and integration of clinical metadata. This includes the symptoms and lab findings, as well as any comorbidities. Multimodal imaging approaches combining chest X-ray, CT, and ultrasound data could further be used to improve diagnostic sensitivity and specificity. Explainable AI techniques such as Grad-CAM, LIME, and SHAP will be significant for fostering clinician trust. They also serve as a guarantee for transparency in automated decision-making. EfficientNet variants demonstrate promise by achieving strong performance with decreased computational demands, thus making them suitable for implementation in both mobile or resource-limited healthcare environments. Ethical considerations, patient privacy, and regulatory compliance must remain the primary focus of future developments to ensure the responsible and fair incorporation of AI into clinical workflows.

## 6. Conclusions

Automated diagnosis based on deep learning has become important in medical imaging, particularly for diseases like COVID-19. In this study, we demonstrate how optimised deep learning architectures combined with strong biomedical signal processing can significantly improve the diagnostic reliability for COVID-19 identification from chest X-ray images. Three models have been studied: CNN, ResNet-50, and EfficientNetB3. ResNet-50 demonstrated the highest classification accuracy (93.7%) and macro-AUC = 0.97. EfficientNetB3 showed superior stability and generalisation across the various data distributions. Preprocessing retained important diagnostic structures, ensuring that model accuracy was not achieved at the expense of clinical information quality, according to signal fidelity analysis (SSIM = 0.93 ± 0.02). The study of model efficiency validated the feasibility of deploying lightweight designs with fewer parameters and rapid convergence. This enabled scalability in real-world radiology processes. Interpretability assessment using Grad-CAM indicated a constant focus on pulmonary opacities and peripheral ground-glass patterns, validating the clinical relevance and transparency of model predictions. This work highlights the critical importance of biological signal and image processing. This provides reliable, easily accessible, and quick computer-assisted diagnosis by combining transfer learning, data balance, and explainable AI. Also, this proposed signal-fidelity-aware framework can be generalized to other medical and multimedia imaging tasks, demonstrating versatility beyond COVID-19 chest X-rays. For future research, this framework will be extended towards multimodal imaging integration and cross-institutional validation, which will further enhance generalization and clinical deployment potential.

## Figures and Tables

**Table 1:** Categorized Literature Review: COVID-19 Detection and Related Works (Sorted by Year Descending).

Category	Author(s)	Year	Model/Architecture/Method	Dataset	Performance/Results	Remarks/Contribution
Image Analysis Techniques	Trigka et al. [2]	2025	Image processing methods (survey)	N/A (Review)	Contrast enhancement, shape refinement	Comprehensive survey of classical and deep learning image processing techniques
	Al-qaness et al. [25]	2024	Thresholding + deep learning	CXR	Survey of preprocessing and DL methods	Review of deep learning techniques for lung disease detection
Classical Machine Learning	Patnaik et al. [6]	2024	Classical ML (SVM)	CXR	Intelligent diagnosis	Machine learning-based COVID-19 diagnosis
	Khekan et al. [3]	2024	Random Forest, KNN	CXR	COVID-19 classification	Evaluation of supervised ML classifiers
Deep Learning	Ayman et al. [8]	2025	CNN architectures	CXR	COVID-19 detection	Multi-class chest disease classification
	Hira et al. [9]	2021	CNN based on ResNeXt50	CXR	97.55% accuracy	Early multi-class COVID-19 classification
	Mienye et al. [4]	2025	CNNs (survey)	N/A (Review)	N/A (Review)	Comprehensive review of CNN applications in medical imaging
Transfer Learning	Kaushik et al. [13]	2024	Transfer learning (ResNet, DenseNet)	Medical images	Improved classification performance	Demonstrates performance gains using pre-trained CNNs
	Khattab et al. [26]	2024	Transfer learning with CNNs	CXR	Automated detection	COVID-19 and pneumonia detection using TL
	Kansal et al. [11]	2024	ResNet-50, EfficientNet-B0	Lung imaging	Multi-class classification	Comparative evaluation of modern CNN architectures
	Mzoughi et al. [10]	2023	EfficientNet (transfer learning)	Chest X-ray	COVID-19 detection	EfficientNet-based transfer learning for chest X-ray classification
Interpretability/ XAI	Patrício et al. [16]	2022	Explainable deep learning (survey)	Medical images	Review	Survey of explainability methods in image classification
	Salvi et al. [20]	2025	Explainability and uncertainty analysis	Medical imaging	Uncertainty-aware interpretability	Examines explainability and uncertainty in deep learning for healthcare

**Table 2:** Summary of hyperparameters for CNN, ResNet-50, and EfficientNetB3.

Parameter	CNN	ResNet-50	EfficientNetB3
Input size	128×128×1	224×224×3	300×300×3
Transfer Learning	No	ImageNet (frozen lower layers)	ImageNet (frozen lower layers)
Epochs	50	15	20
Batch size	32	32	8
Optimizer	Adam	Adam	Adam
Learning rate	0.001	0.0001	Adaptive scheduling
Regularization	Dropout, Early stopping	Early stopping	Dropout, Augmentation, Early stopping
Fine-tuned layers	None	Upper dense layers (4 classes)	Custom head with BatchNorm, Dropout, Dense
Dataset variants	Unbalanced, Undersampled, Oversampled, SMOTE	Unbalanced, Undersampled, Oversampled	Unbalanced only

**Table 3:** Quantitative Explainability (XAI) evaluation across models.

Model	SSIM ↑	IoU ↑	Dice ↑	Ins/Del AUC ↑
CNN	0.91	0.72	0.77	0.84
ResNet-50	0.93	0.76	0.81	0.88
EfficientNetB3	0.94	0.78	0.83	0.90

**Table 4:** Test accuracy (%) of CNN, ResNet-50, and EfficientNetB3 across dataset variants.

Dataset Variant	CNN (%)	ResNet-50 (%)	EfficientNetB3 (%)
Unbalanced	85.0	91.7	88.0
Undersampled	82.0	89.8	N/A
Oversampled	87.0	93.7	N/A
SMOTE-balanced	85.0	N/A	N/A

## Data Availability

The data presented in this study are openly available in COVID-19 Radiography Dataset at https://www.kaggle.com/datasets/tawsifurrahman/covid19-radiography-database (accessed on 21 December 2025).

## References

[1] Bhatele K.R., Jha A., Tiwari D., Bhatele M., Sharma S., Mithora M.R., Singhal S. (2024). COVID-19 detection: A systematic review of machine and deep learning-based approaches utilizing chest X-rays and ct scans. Cogn. Comput..

[2] Trigka M., Dritsas E. (2025). A Comprehensive Survey of Deep Learning Approaches in Image Processing. Sensors.

[3] Khekan A.R. (2024). Classification and Evaluation of the COVID-19 Dataset Based on Supervised Machine Learning Techniques. IAR J. Eng. Technol..

[4] Mienye I.D., Swart T.G., Obaido G., Jordan M., Ilono P. (2025). Deep convolutional neural networks in medical image analysis: A review. Information.

[5] Muhammad D., Ahmed I., Ahmad M.O., Bendechache M. (2025). Randomized Explainable Machine Learning Models for Efficient Medical Diagnosis. IEEE J. Biomed. Health Inform..

[6] Patnaik A., Krishna P.K. (2024). Intelligent decision support system in healthcare using machine learning models. Recent Patents Eng..

[7] Vangipuram S.K., Appusamy R. (2025). A Novel Image Feature Extraction Based Machine Learning approach for Disease Detection from Chest X-Ray Images. J. Electron. Electromed. Eng. Med. Inform..

[8] Ayman N., Gadallah M.E., Saeid M.M. (2025). Multi-Classification Convolution Neural Network Models for Chest Disease Classification. Int. J. Adv. Comput. Sci. Appl..

[9] Hira S., Bai A., Hira S. (2021). An automatic approach based on CNN architecture to detect COVID-19 disease from chest X-ray images. Appl. Intell..

[10] Mzoughi H., Njeh I., Slima M.B., BenHamida A. (2023). Deep efficient-nets with transfer learning assisted detection of COVID-19 using chest X-ray radiology imaging. Multimed. Tools Appl..

[11] Kansal K., Chandra T.B., Singh A. (2024). ResNet-50 vs. EfficientNet-B0: Multi-Centric Classification of Various Lung Abnormalities Using Deep Learning “Session id: ICMLDsE. 004”. Procedia Comput. Sci..

[12] Haynes S.C., Johnston P., Elyan E. (2024). Generalisation challenges in deep learning models for medical imagery: Insights from external validation of COVID-19 classifiers. Multimed. Tools Appl..

[13] Kaushik P., Khan Z., Kajla A., Verma A., Khan A. (2024). Enhancing object recognition with resnet-50: An investigation of the cifar-10 dataset. 2023 International Conference on Smart Devices (ICSD).

[14] Rasheed S. (2026). Lightweight Deep Learning Models for Face Mask Detection in Real-Time Edge Environments: A Review and Future Research Directions. Preprints.

[15] Yaqoub K.Y., Abdulazeez A.M. (2024). Empowering Diagnosis: A Review On Deep Learning Applications for COVID-19 and Pneumonia in X-Ray Images. JISA (J. Inform. Dan Sains).

[16] Patrício C., Neves J.C., Teixeira L.F. (2022). Explainable Deep Learning Methods in Medical Image Classification: A Survey. arXiv.

[17] Chaddad A., Hu Y., Wu Y., Wen B., Kateb R. (2025). Generalizable and explainable deep learning for medical image computing: An overview. Curr. Opin. Biomed. Eng..

[18] Singh Y., Hathaway Q.A., Keishing V., Salehi S., Wei Y., Horvat N., Vera-Garcia D.V., Choudhary A., Mula Kh A., Quaia E. (2025). Beyond Post hoc Explanations: A Comprehensive Framework for Accountable AI in Medical Imaging Through Transparency, Interpretability, and Explainability. Bioengineering.

[19] Sharma S., Singh M., McDaid L., Bhattacharyya S. (2025). XAI-based data visualization in multimodal medical data. bioRxiv.

[20] Salvi M., Seoni S., Campagner A., Gertych A., Acharya U.R., Molinari F., Cabitza F. (2025). Explainability and uncertainty: Two sides of the same coin for enhancing the interpretability of deep learning models in healthcare. Int. J. Med. Inform..

[21] Ennab M., Mcheick H. (2025). Advancing AI interpretability in medical imaging: A comparative analysis of pixel-level interpretability and Grad-CAM models. Mach. Learn. Knowl. Extr..

[22] Rundo L., Militello C. (2024). Image biomarkers and explainable AI: Handcrafted features versus deep learned features. Eur. Radiol. Exp..

[23] Kinger S., Kulkarni V. (2025). Transparent and trustworthy interpretation of COVID-19 features in chest X-rays using explainable AI. Multimed. Tools Appl..

[24] Tang H., Li Z., Zhang D., He S., Tang J. (2024). Divide-and-conquer: Confluent triple-flow network for RGB-T salient object detection. IEEE Trans. Pattern Anal. Mach. Intell..

[25] Al-qaness M.A., Zhu J., AL-Alimi D., Dahou A., Alsamhi S.H., Abd Elaziz M., Ewees A.A. (2024). Chest x-ray images for lung disease detection using deep learning techniques: A comprehensive survey. Arch. Comput. Methods Eng..

[26] Khattab R., Abdelmaksoud I.R., Abdelrazek S. (2024). Automated detection of COVID-19 and pneumonia diseases using data mining and transfer learning algorithms with focal loss from chest X-ray images. Appl. Soft Comput..

[27] Rahman T. (2020). COVID-19 Radiography Database. https://www.kaggle.com/datasets/tawsifurrahman/covid19-radiography-database.

[28] Borys K., Schmitt Y.A., Nauta M., Seifert C., Krämer N., Friedrich C.M., Nensa F. (2023). Explainable AI in medical imaging: An overview for clinical practitioners–Beyond saliency-based XAI approaches. Eur. J. Radiol..

